# Brazilian Portuguese translation and cross-cultural adaptation of the “Caregiver Priorities and Child Health Index of Life with Disabilities” (CPCHILD^©^) questionnaire

**DOI:** 10.1186/1471-2431-14-30

**Published:** 2014-02-01

**Authors:** Luiz Antonio Pellegrino, Erika Veruska Paiva Ortolan, Claudia Saad Magalhães, Ariane Aparecida Viana, Unni G Narayanan

**Affiliations:** 1 Bauru State Hospital Bauru, Bauru, SP ZIP 17033-360, Brazil; 2 Department of Surgery and Orthopedics, Post Graduate Surgery Program, Botucatu Medical School, Botucatu, SP ZIP 18618-970, Brazil; 3 Department of Pediatrics and Pediatric Rheumatology, Post Graduate Public Health Program, Botucatu Medical School, Botucatu, SP ZIP 18618-970, Brazil; 4 Divisions of Orthopaedic Surgery & Child Health Evaluative Sciences, The Hospital for Sick Children, Bloorview Research Institute, University of Toronto, Toronto, Ontario M5G 1X8, Canada

**Keywords:** Translation, Adaptation, Questionnaire, Assessment, Health-related quality of life, Developmental disabilities

## Abstract

**Background:**

Consideration of the quality of life in relation to individual health status is crucial for planning and maintaining a system of patient-centered care. Until recently, there have been no suitable instruments to assess health-related quality of life (HRQoL,) of children and adolescents with severe, non-ambulant cerebral palsy (GMFCS functional levels IV and V). The “Caregiver Priorities and Child Health Index of Life with Disabilities Questionnaire” (CPCHILD^©^) was developed in English specifically for this population, and has been validated in Canada. The aim of this study was to translate and adapt the CPCHILD^©^ Questionnaire into Brazilian Portuguese, thus permitting researchers in Brazil to access this important tool for measuring HRQoL in this population, as well as the possibility of making comparisons with other studies that use the same questionnaire in other languages.

**Method:**

The cross-cultural adaptation included two forward translations by independent translators, their synthesis, two back-translations by independent translators, an assessment of the versions by an expert committee and the development of a pre-final version, which was tested on 30 caregivers of children (5 -18) with severe cerebral palsy (GMFCS IV & V).

**Results:**

Despite the relative equivalence between the two translations, some items required adaptations for the synthesized version. Certain modifications were necessary in the pre-final version to achieve idiomatic equivalence. The modifications were required to account for the socioeconomic and cultural levels of the target population.

**Conclusion:**

The translation and cross-cultural adaptation of the CPCHILD^©^ questionnaire provides a Brazilian Portuguese equivalent to measure the HRQoL of children with severe developmental disabilities, with the potential to measure the benefits of various procedures that are indicated for these patients. This adaptation exhibited a satisfactory level of semantic equivalence between the Portuguese target and the original English source versions. The validity of the Brazilian version of the instrument must be established in the future by assessing its psychometric properties on Brazilian epidemiological samples.

## Background

Cerebral palsy (CP) is one of the most common causes of chronic disability in childhood; with an incidence of 2 to 2.5 per 1.000 live births reported in developed countries [1-3]. In Brazil, 24.5 million people present some kind of disability, including children with cerebral palsy [4]. Brazilian studies concerning the frequency of this disorder are nonexistent, despite an estimated 30.000 and 40.000 new cases per year [5]. Although the primary brain lesion is not progressive, the pathological condition is permanent, and many of the clinical manifestations of CP, including musculoskeletal effects, are acquired and progress over time. Between 25% and 35% of affected children are severely affected and they exhibit health problems, difficulties with everyday life activities, communication, mobility, and are dependent on caregivers for the majority of their needs. These circumstances strongly impact the patients’ lives, the lives of their caregivers and relatives and the institutions that are responsible for their care and wellbeing [6-8].

Health-related quality of life (HRQoL) is an important consideration in the planning and maintenance of a patient-centered health care system [9]. However, measuring HRQoL of children and adolescents with severe developmental disabilities (Gross Motor Function Classification System – GMFCS – functional levels IV and V [10]) is challenging and instruments for these measurements are scarce. There are several generic and condition-specific QoL instruments available. These instruments are often developed with minimal involvement of families, focusing on functional abilities rather than on well-being, and contain items that are negatively worded [11]. Generic instruments for health status assessments, such as the *Child Health Questionnaire (CHQ)*[12] and the *Pediatric Evaluation of Disability Inventory (PEDI)*[13], do not accurately measure the HRQoL of individuals with severe CP. The CP module of the Pediatric Quality of Life Inventory (PedsQL-CP module), a condition-specific HRQoL questionnaire for children and adolescents with CP, demonstrated that children with quadriplegia presented significantly lower HRQoL than children with hemiplegia and diplegia, and children with minimal functional difficulty on the GMFCS demonstrated significantly higher HRQoL [14]. However, researchers and clinicians should be aware of how an instrument was developed and its theoretical focus, as these will influence the domains, items, and resulting scores [11]. A disease-specific measure of health status and well-being of children with severe cerebral palsy, the *Caregiver Priorities and Child Health Index of Life with Disabilities* (CPCHILD^©^) [15] was developed specifically for this population. Besides reviewing other questionnaires, the CPCHILD^©^ instrument was developed based on the recommendations of caregivers of children with severe cerebral palsy, as well as input from healthcare professionals with experience in treating this population. The questionnaire was developed in English for a Canadian population. Given the increased frequency of multinational and multicultural research projects and the lack of a questionnaire for CP children translated into Brazilian Portuguese, the need to translate and adapt health status measures to enable the comparison of results across cultures continues to grow [16]. The objective of this study was to translate and adapt the CPCHILD^©^ questionnaire to Brazilian Portuguese.

## Methods

The study was conducted at the Rehabilitation Unit of Bauru State Hospital, which is part of the Botucatu Medical School of São Paulo State University. The study was approved by the Research Ethics Committee of the Botucatu Medical School.

Parents who were the primary caregivers of children with cerebral palsy (GMFCS functional levels I to V) aged five to 18-years old were included. All the participants signed a term of free informed consent. Caregivers of patients with diagnoses other than cerebral palsy and caregivers who refused to participate were excluded.

The author of the source questionnaire written in English granted permission for its use, translation, cultural adaptation and validation in Brazilian Portuguese.

### Translation and cross-cultural adaptation

The adaptation of health status questionnaires for new countries, cultures or languages requires a unique method to achieve equivalence between the original source and target versions. For this reason, the CPCHILD^©^ instrument was submitted to translation and cross-cultural adaptation according to internationally accepted and recommended guidelines [16-18], which are currently used by the *American Association of Orthopaedic Surgeons* (AAOS) *Outcomes Committee* (Additional file 1: Figure S1) [18].

In Stage 1, the source version of the CPCHILD Questionnaire was translated separately by two independent translators (T1 and T2). In Stage 2, the two resulting translations (A1 and A2) were reviewed by an expert committee that included the two translators, the principal investigator, the supervisor, and a professor with a PhD and experience in the cross-cultural adaptation of quality of life instruments for children. The team members who voted for the best option of each line then disclosed their choice, and a discussion, including the translators, continued until consensus was achieved.

The two translators (BT1 & BT2) at Stage 3 (back-translation) did not participate in the first stage. The back-translations (B1 and B2) were entirely independent and were performed without knowledge of the source version of the questionnaire.

To develop the pre-final version, the B1 and B2 back-translations of the questionnaire were compared with the source version. The role of the committee was to consolidate the versions of the questionnaire (A1, A2, A-12, B1, and B2), and to develop the pre-final version. Decisions were made by the committee to achieve equivalence between the components of the translated and source versions [17].

The pre-final version was applied by the main author to 30 caregivers, without interpretations [18]. The scores were calculated according to rules that were established by Narayanan et al. [15], which indicate that at least 50% of the items of each scale must be answered to generate a score. After completing the pre-final version of the questionnaire, the participants were interviewed to assess their understanding of the items in order to identify other comprehension problems. According to the method proposed by Ciconelli et al. [19], when more than 15% of the participants express doubt or a lack of understanding regarding an item, the item must be reviewed by the expert committee and reapplied to other participants.

Stage 6 involved assessments of the pre-test results and the caregiver comments. The final version was developed after a discussion of the items that remained difficult to understand.

The final version of the instrument translated and adapted for Brazilian Portuguese can be downloaded at: http://www.sickkids.ca/Research/CPCHILD-Questionaire/CPCHILD-Project/CPChild-questionaire/index.html.

## Results

After the English source version had been translated to Portuguese in Stage 1, the team concluded that despite the relative equivalence between the two translations, certain items required adaptation to develop the synthesized version (A-12). Specifically, the translations exhibited significant differences in the general scaling of the child’s level of education, which is included in the demographic section of the source questionnaire. Thus, consensus was impossible because the criteria were different. To standardize the educational levels, the system that is currently used by the Brazilian Ministry of Education was selected.

To develop the pre-final version, the expert committee reached consensus regarding several questions, and certain items were modified where conceptual equivalence was possible. These modifications were necessary to achieve idiomatic equivalence, while considering the socioeconomic and cultural levels of the target population. For some items, one version was prioritized; for other items, both versions were combined. The meanings of terms that best approximated the source items were included.

After the synthesized version was created and the pre-test was performed, several items required further adaptation for the final version. These adaptations were based on the participating professionals’ experiences and the suggestions and doubts that were expressed by the participants during the interview following the pre-final version. Terms and sentences that were difficult to understand were modified. The modifications generated at different points in the adaptation process are summarized in Additional file 2: Table S1. The sample of caregivers consisted of females between 24 and 59 years of age (mean 40.43 ± 8.41 years-old). The predominant educational level of the caregivers was elementary school (63.33%), followed by middle school (26.67%), technical education (6.67%), and higher education (3.33%). The average time to complete the questionnaire was 28 ± 12.33 minutes, and individuals with lower educational levels required more time to complete the questionnaire. The majority of the caregivers understood that the questionnaire was designed to investigate the health, comfort, wellbeing, and care of their children.

The final version of the instrument translated and adapted for Brazilian Portuguese can be viewed at Additional file 3 or downloaded at http://www.sickkids.ca/Research/CPCHILD-Questionaire/CPCHILD-Project/CPChild-questionaire/index.html.

## Discussion

There is agreement regarding the importance of assessing the quality of life of patients with developmental disabilities, particularly cerebral palsy [20]. These assessments can guide therapeutic decision-making toward areas that are important for patients and their caregivers [21] and such instruments can be applied in clinical practice to assess the effects of therapeutic interventions on actual improvements in health-related quality of life.

Although the objectives of most therapeutic interventions are to preserve and improve the quality of life of patients, they are frequently associated with high costs and cause stress to patients and their relatives. In addition, tangible evidence of the benefits of intervention is not always available [22].

Therefore, adequate measures of quality of life can identify individual priorities that can align therapeutic goals with the actual needs of patients and caregivers [9,21,23,24].

Narayanan et al. [25] established that the CPHILD^©^ was sensitive in a cohort of children with CP undergoing scoliosis surgery and was able to measure significant improvements at 6 and 12 months postsurgery. The authors concluded that CPCHILD^©^ can be used as a meaningful outcome measure of the effectiveness of interventions for scoliosis in children with severe cerebral palsy.

Studies on the cross-cultural adaptation of HRQoL instruments for patients with severe cerebral palsy in Brazil have not been previously conducted. Given the relevance of these instruments for professionals who assist patients with cerebral palsy and for related public health policies, the translation and cross-cultural adaptation of the CPCHILD^©^ provides objective information concerning the benefits of various procedures that are indicated for these patients.

The use of detailed methods, such as those proposed by Beaton et al. [16], was important to identify the primary difficulties in the development of equivalent versions.

This study represents the first step in the cross-cultural adaptation of the CPCHILD^©^ instrument. The second step is to validate the instrument’s psychometric properties in Brazilian epidemiological samples. The same research team has already performed this step and are currently at the stage of data collection using a much larger sample of parents and caregivers of children with cerebral palsy (functional levels I to V). The results will consolidate and validate the instrument for the Brazilian population.

## Conclusions

The translation and cross-cultural adaptation of the CPCHILD^©^ questionnaire provides a Brazilian Portuguese version that can measure the health-related quality of life of children with severe developmental disabilities, with the potential to measure the actual benefits of various procedures that are indicated for patients with developmental disabilities. This adaptation exhibited a satisfactory level of semantic equivalence between the Portuguese target and the original English source versions.

## Competing interests

The authors declare that they have no competing interests.

## Authors’ contributions

LAP conceived the study, participated in its design, provided substantial contributions to background analysis and literature research, and drafted the manuscript. EVPO provided methodological guidance and contributed to discussions regarding analyses and interpretation of the results, and revised the manuscript. CSM provided substantial contributions to the design of the study and was member of the expert committee. AVV provided substantial contributions to data acquisition, parents’ interviews, development of the database, and helped to draft the manuscript. UGN provided substantial contributions to the conception and design of the study, and revised the manuscript. All the authors read and approved the final manuscript.

## Pre-publication history

The pre-publication history for this paper can be accessed here:

http://www.biomedcentral.com/1471-2431/14/30/prepub

## Supplementary Material

Additional file 1: Figure S1 Summary of the translation and cross-cultural adaptation guidelines. Click here for file

Additional file 2: Table S1 Short description of the modifications made to the synthesized version, pre-final version and final version. Click here for file

Additional file 3 **Final version of the CPCHILD**^**©**^** instrument translated and adapted for Brazilian Portuguese.** Click here for file
