# Supplementary figures and images for: Brazilian Portuguese translation and cross-cultural adaptation of the “Caregiver Priorities and Child Health Index of Life with Disabilities” (CPCHILD©) questionnaire
Source: BMC Pediatr. 2014 Feb 1;14:30. doi: 10.1186/1471-2431-14-30 (PMC3915619; doi:10.1186/1471-2431-14-30)

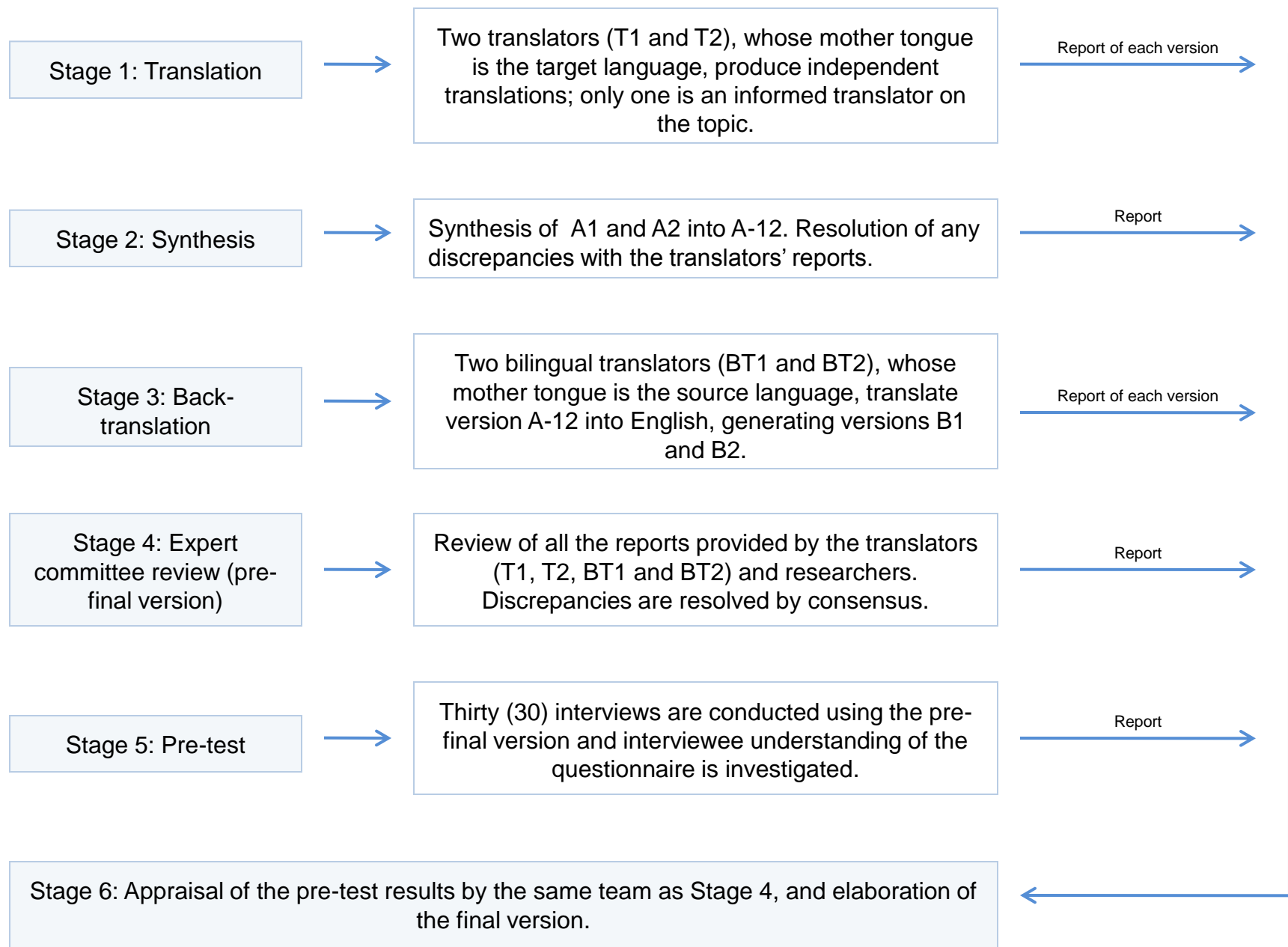

Supplement: Additional file 1: Figure S1 — Summary of the translation and cross-cultural adaptation guidelines. [file 1471-2431-14-30-S1.pdf]
