# Supplementary material for: Brazilian Portuguese translation and cross-cultural adaptation of the “Caregiver Priorities and Child Health Index of Life with Disabilities” (CPCHILD©) questionnaire
Source: BMC Pediatr. 2014 Feb 1;14:30. doi: 10.1186/1471-2431-14-30 (PMC3915619; doi:10.1186/1471-2431-14-30)
Supplement: Additional file 2: Table S1 — Short description of the modifications made to the synthesized version, pre-final version and final version. [file 1471-2431-14-30-S2.pdf]

Section / Item	Synthesis version		Pre-final version		Final version	
	Discussion	Consensus	Discussion	Consensus	Discussion	Consensus
Title	▪ Symbol *&*<br>▪ *Infantil*<br>▪ *Convivendo com deficiência*	▪ Substituted for *e*<br>▪ *Infantil* was removed<br>▪ Changed to *criança com deficiências*	▪ Inclusion of the term *convivendo com deficiências*	▪ The T1 original translation was maintained		
-					▪ *Formulário*	▪ *Questionário*
Instruction / 1	▪ your child's health	▪ Changed to *seu filho(a)*				
Instruction / 2	▪ *Leia atentamente ou cuidadosamente*	▪ *Leia as instruções com atenção*	▪ Use of the term *por favor*	▪ The term was removed		
Instruction / Example	▪ Level of assistance<br>▪ Minimal supervised<br>▪ Putting on/wearing footwear? (socks, shoes, braces, etc.)	▪ *Nível de Ajuda*<br>▪ *Mínimo ou Supervisionado*<br>▪ *Colocar meias, sapatos, aparelho ortopédico, etc.*				
1 / 1			▪ *Da maneira como isso normalmente é feito*	▪ Substituted for *da maneira usual*	▪ The phrase: *comer/beber ou ser alimentado?*	▪ *Alimentar seu filho(a)?*
1 / 3	▪ bathing/washing	▪ *Tomar banho*	▪ bathing/washing	▪ *banhar/lavar* was maintained	▪ bathing/washing	▪ *banhar/lavar* was maintained
1 / 7	▪ *Desvestir-se*	▪ Changed to *tirar roupas*				
2 / 10			▪ *Deitar-se ou levantar-se da cama*	▪ *Deitar e levantar da cama*		
2 / 11	▪ Transferring into/out of a wheelchair/chair?	▪ *Transferir-se para ou de uma cadeira de rodas ou cadeira*				
2 / 12					▪ *Sentar*	▪ *Permanecer sentado(a)*
2 / 13			▪ Standing up	▪ Changed to *em pé*		
2 / 14 and 15					▪ *Movimentar-se*	▪ *Locomover-se*
2 / 17	▪ Sightseeing	▪ *Excursões*				
3 / -			▪ *Forte* in the T12 version generated the term strong and not severe as in the original	▪ The term *intensidade* was altered to *grau* and the terms *forte* to *intenso* and *fraca* to *leve*	▪ Inclusion of a statement	▪ Insert the statement
4 / 32					▪ Inclusion of the word *alfabetização*	▪ Include the word *alfabetização*
5 / 3			▪ *Ruim* and *muito ruim* generated the term bad and very bad and not poor and very poor	▪ Maintain the original translation		
5 / 34					▪ *Internado > 7 dias*<br>▪ *Internado < 7 dias*	▪ *Ficou internado menos que/por mais de uma semana*
7 / -					▪ The option: *razoavelmente importante*<br>▪ The option: *muito importante*	▪ *Mais ou menos importante*<br>▪ *Importante*
7 / 4					▪ The phrase: *Usar o banheiro ou higiene*	▪ *Usar o banheiro ou fazer a higiene*
7 / 12					▪ The word: *Sentar*	▪ *Permanecer sentado(a)*
7 / 17 e 15					▪ The word: *Movimentar-se*	▪ *Locomover-se*
7 / 32					▪ Inclusion of the word *alfabetização*	▪ Include the word *alfabetização*
8 / 1			▪ *Sexo da criança*	▪ The term was maintained		
8 / Level of schooling	▪ Impossible to achieve consensus due to different criteria	▪ *MEC* scale				
9 / 3			▪ *Trabalhando fora em tempo integral ou meio período, ou trabalhando em casa (negócio montado em casa)*	▪ *Trabalhando em tempo integral ou meio período, em casa ou fora*	▪ *Trabalhando em tempo integral ou meio período, em casa ou fora*	▪ *Trabalho em tempo integral ou meio período, em casa ou fora*
Final question	▪ in units of time	▪ *em minutos*				
-					▪ *Nível de auxílio*	▪ Substituted for *nível de ajuda*
-					▪ *Moderado*	▪ Substitute for *médio*
