# Supplementary material for: Brazilian Portuguese translation and cross-cultural adaptation of the “Caregiver Priorities and Child Health Index of Life with Disabilities” (CPCHILD©) questionnaire
Source: BMC Pediatr. 2014 Feb 1;14:30. doi: 10.1186/1471-2431-14-30 (PMC3915619; doi:10.1186/1471-2431-14-30)
Supplement: Additional file 3 — Final version of the CPCHILD © instrument translated and adapted for Brazilian Portuguese. [file 1471-2431-14-30-S3.pdf]

Versão final do questionário CPCHILD© traduzido e adaptado para a língua portuguesa no Brasil

Prioridades do Cuidador e Índice de Saúde da Criança com Deficiências

Instruções

1. Este questionário é sobre a saúde, conforto e bem-estar do seu filho(a), e o cuidado no atendimento de suas necessidades.

2. Leia as instruções com atenção.

3. Responda todas as perguntas fazendo um círculo em volta do número que corresponde à melhor resposta. Você pode acrescentar comentários ou esclarecimentos no espaço deixado em baixo de cada pergunta.

NÍVEL DE AJUDA

<table>
<tr><td colspan="8">Por exemplo:

Considere como cada uma das atividades a seguir é <u>geralmente</u> realizada por/para seu filho(a). Avalie o <u>grau de dificuldade</u> de cada uma dessas atividades nas últimas 2 semanas e indique o <u>nível de ajuda</u> que foi necessária para ajudar seu filho(a) a realizá-las.</td><td rowspan="2">TOTAL</td><td rowspan="2">MÉDIO</td><td rowspan="2">MÍNIMO OU SUPERVISIONADO</td><td rowspan="2">NENHUM</td></tr>
<tr><td>Nas últimas <u>duas semanas</u>, qual foi o grau de dificuldade nas seguintes situações?</td><td>Impossível</td><td>Muito Difícil</td><td>Difícil</td><td>Um Pouco Difícil</td><td>Fácil</td><td>Muito Fácil</td><td>Sem Nenhum Problema</td></tr>
<tr><td>1. Colocar meias, sapatos, aparelho ortopédico, etc.?</td><td>0</td><td>1</td><td>2</td><td>3</td><td>4</td><td>5</td><td>6</td><td>0</td><td>1</td><td>2</td><td>3</td></tr>
</table>

No exemplo acima, a tarefa de colocar meias, sapatos, aparelhos ortopédicos, etc. foi considerada *muito fácil,* e a criança exigiu um nível de auxílio *mínimo ou supervisionado* para realizá-la.

4. Ao final de cada seção, há um espaço para você adicionar quaisquer outros itens, que na sua opinião, estejam faltando no questionário e que você julgue importante para a saúde, conforto e bem-estar de seu filho(a).

Nome da criança:..

Nome do pai/mãe ou cuidador(a) que preencheu este questionário:...

Data:........../........../..........

SEÇÃO 1: CUIDADOS PESSOAIS / ATIVIDADES DA VIDA DIÁRIA

NÍVEL DE AJUDA

Considere como cada uma das atividades a seguir é **<u>geralmente</u>** realizada por/para seu filho(a). Avalie o **<u>grau de dificuldade</u>** de cada uma dessas atividades nas últimas 2 semanas **e** indique o **<u>nível de ajuda</u>** que foi necessária para ajudar seu filho(a) a realizá-las.								TOTAL	MÉDIO	MÍNIMO OU SUPERVISIONADO	NENHUM
**Nas últimas <u>duas semanas</u>, qual foi o grau de dificuldade nas seguintes situações:**	*Impossível*	*Muito Difícil*	*Difícil*	*Um Pouco Difícil*	*Fácil*	*Muito Fácil*	*Sem Nenhum Problema*				
**1. Alimentar seu filho(a)? (da maneira usual, por exemplo, oralmente, por sonda ou ambos).**	**0**	**1**	**2**	**3**	**4**	**5**	**6**	**0**	**1**	**2**	**3**
**2. Manter a higiene bucal? (manutenção da boca e dos dentes limpos).**	**0**	**1**	**2**	**3**	**4**	**5**	**6**	**0**	**1**	**2**	**3**
**3. Dar banho em seu filho(a)?**	**0**	**1**	**2**	**3**	**4**	**5**	**6**	**0**	**1**	**2**	**3**
**4. Utilizar o banheiro? (urinar, defecar e limpar-se).**	**0**	**1**	**2**	**3**	**4**	**5**	**6**	**0**	**1**	**2**	**3**
**5. Trocar fraldas (roupas de baixo)?**	**0**	**1**	**2**	**3**	**4**	**5**	**6**	**0**	**1**	**2**	**3**
**6. Vestir ou tirar roupas da parte de cima do corpo? (camisa, casaco, etc.).**	**0**	**1**	**2**	**3**	**4**	**5**	**6**	**0**	**1**	**2**	**3**
**7. Vestir ou tirar roupas da parte de baixo do corpo? (calças, abrigo, etc.).**	**0**	**1**	**2**	**3**	**4**	**5**	**6**	**0**	**1**	**2**	**3**
**8. Colocar meias, sapatos, aparelho ortopédico, etc.?**	**0**	**1**	**2**	**3**	**4**	**5**	**6**	**0**	**1**	**2**	**3**

**9. Cuidar dos cabelos? (lavar, secar, escovar, pentear, trançar, etc.).**	**0**	**1**	**2**	**3**	**4**	**5**	**6**	**0**	**1**	**2**	**3**
**1A. Outros cuidados pessoais? Especifique:**	**0**	**1**	**2**	**3**	**4**	**5**	**6**	**0**	**1**	**2**	**3**
**1B. Outros cuidados pessoais? Especifique:**	**0**	**1**	**2**	**3**	**4**	**5**	**6**	**0**	**1**	**2**	**3**

SEÇÃO 2: POSICIONAMENTO, TRANSFERÊNCIA E MOBILIDADE

NÍVEL DE AJUDA

Considere como cada uma das atividades a seguir é **geralmente** realizada por/para seu filho(a). Avalie o **grau de dificuldade** de cada uma dessas atividades nas últimas 2 semanas **e** indique o **nível de ajuda** que foi necessária para ajudar seu filho(a) a realizá-las.								TOTAL	MÉDIO	MÍNIMO OU SUPERVISIONADO	NENHUM
**Nas últimas duas semanas, qual foi o grau de dificuldade nas seguintes situações:**	*Impossível*	*Muito Difícil*	*Difícil*	*Um Pouco Difícil*	*Fácil*	*Muito Fácil*	*Sem Nenhum Problema*				
**10. Deitar e levantar da cama?**	**0**	**1**	**2**	**3**	**4**	**5**	**6**	**0**	**1**	**2**	**3**
**11. Transferir para ou de uma cadeira de rodas ou cadeira?**	**0**	**1**	**2**	**3**	**4**	**5**	**6**	**0**	**1**	**2**	**3**
**12. Permanecer sentado(a) na cadeira de rodas ou cadeira?**	**0**	**1**	**2**	**3**	**4**	**5**	**6**	**0**	**1**	**2**	**3**
**13. Manter-se em pé para se exercitar ou mudar de lugar?**	**0**	**1**	**2**	**3**	**4**	**5**	**6**	**0**	**1**	**2**	**3**

**14. Locomover-se pela casa? (do jeito que for possível).**	**0**	**1**	**2**	**3**	**4**	**5**	**6**	**0**	**1**	**2**	**3**
**15. Locomover-se fora de casa? (do jeito que for possível).**	**0**	**1**	**2**	**3**	**4**	**5**	**6**	**0**	**1**	**2**	**3**
**16. Entrar e sair de veículos? (carro, van ou ônibus).**	**0**	**1**	**2**	**3**	**4**	**5**	**6**	**0**	**1**	**2**	**3**
**17. Ir a lugares públicos? (praças, cinema, teatros, excursões, etc.).**	**0**	**1**	**2**	**3**	**4**	**5**	**6**	**0**	**1**	**2**	**3**
**2A. Outra atividade? Especifique:**	**0**	**1**	**2**	**3**	**4**	**5**	**6**	**0**	**1**	**2**	**3**
**2B. Outra atividade? Especifique:**	**0**	**1**	**2**	**3**	**4**	**5**	**6**	**0**	**1**	**2**	**3**

SEÇÃO 3: CONFORTO E EMOÇÕES

GRAU

Nas últimas 2 semanas, considere se durante as atividades a seguir seu filho(a) teve dor ou desconforto. Avalie a **frequência** e o **grau** da dor ou desconforto.							INTENSO	MÉDIO	LEVE	NENHUM
**Nas últimas duas semanas, com que frequência seu filho(a) teve dor ou desconforto:**	*Todos os dias*	*Com muita frequência*	*Com frequência regular*	*Algumas vezes*	*Uma ou duas vezes*	*Nenhuma vez*				
**18. Ao comer ou beber ou ser alimentado?**	**0**	**1**	**2**	**3**	**4**	**5**	**0**	**1**	**2**	**3**
**19. Durante o uso do banheiro? (urinar, defecar, limpar-se, trocar fraldas, etc.).**	**0**	**1**	**2**	**3**	**4**	**5**	**0**	**1**	**2**	**3**
**20. Enquanto colocava ou tirava roupas?**	**0**	**1**	**2**	**3**	**4**	**5**	**0**	**1**	**2**	**3**
**21. Ao se transferir de um lugar para outro ou mudar de posição?**	**0**	**1**	**2**	**3**	**4**	**5**	**0**	**1**	**2**	**3**
**22. Na posição sentada?**	**0**	**1**	**2**	**3**	**4**	**5**	**0**	**1**	**2**	**3**

**23. Deitado(a) na cama?**	**0**	**1**	**2**	**3**	**4**	**5**	**0**	**1**	**2**	**3**
**24. Que perturbou o sono de seu filho(a)?**	**0**	**1**	**2**	**3**	**4**	**5**	**0**	**1**	**2**	**3**
**3A. Durante outra atividade? Especifique:**	**0**	**1**	**2**	**3**	**4**	**5**	**0**	**1**	**2**	**3**
**3B. Durante outra atividade? Especifique:**	**0**	**1**	**2**	**3**	**4**	**5**	**0**	**1**	**2**	**3**
							**GRAU**			
**Nas últimas <u>duas semanas</u>, com que frequência seu filho(a) ficou:**	*Todos os dias*	*Com muita frequência*	*Com frequência regular*	*Algumas vezes*	*Uma ou duas vezes*	*Nenhuma vez*	INTENSO	MÉDIO	LEVE	NENHUM
**25. Agitado(a), aborrecido(a) ou bravo(a)?**	**0**	**1**	**2**	**3**	**4**	**5**	**0**	**1**	**2**	**3**
**26. Infeliz ou triste?**	**0**	**1**	**2**	**3**	**4**	**5**	**0**	**1**	**2**	**3**

SEÇÃO 4: COMUNICAÇÃO E INTERAÇÃO SOCIAL

Considere como cada uma das atividades a seguir é **<u>geralmente</u>** realizada por/para seu filho(a). Classifique o **<u>grau de dificuldade</u>** de cada uma dessas atividades nas últimas 2 semanas.

**Nas últimas <u>duas semanas</u>, qual foi o grau de dificuldade que seu filho(a) teve para:**	*Impossível*	*Muito Difícil*	*Difícil*	*Um Pouco Difícil*	*Fácil*	*Muito Fácil*	*Sem Nenhum Problema*
**27. Compreender você?**	**0**	**1**	**2**	**3**	**4**	**5**	**6**
**28. Ser compreendido por você?**	**0**	**1**	**2**	**3**	**4**	**5**	**6**
**29. Na comunicação com pessoas que não conhecem bem seu filho(a)?**	**0**	**1**	**2**	**3**	**4**	**5**	**6**

**30. Brincar sozinho(a)?**	**0**	**1**	**2**	**3**	**4**	**5**	**6**
**31. Brincar com outras pessoas?**	**0**	**1**	**2**	**3**	**4**	**5**	**6**
**32. Frequentar a escola, a creche ou a alfabetização?**	**0**	**1**	**2**	**3**	**4**	**5**	**6**
**33. Participar em atividades recreativas (nadar, interagir com a família e amigos, etc.)?**	**0**	**1**	**2**	**3**	**4**	**5**	**6**
**4A. Outra atividade social?**<br>**Especifique:**	**0**	**1**	**2**	**3**	**4**	**5**	**6**
**4B. Outra atividade social?**<br>**Especifique:**	**0**	**1**	**2**	**3**	**4**	**5**	**6**

SEÇÃO 5: SAÚDE

**Nas últimas <u>duas semanas</u>**	**Faça um círculo ao redor da resposta mais apropriada**					
**34. Quantas vezes seu filho(a) teve que ir ao médico ou ao hospital?**	*Ficou internado por mais de 1 semana*	*Ficou internado menos de 1 semana*	*3 ou mais vezes*	*Duas vezes*	*Uma vez*	*Nenhuma vez*
	**0**	**1**	**2**	**3**	**4**	**5**
**35. Como você classificaria a saúde geral de seu filho(a)?**	*Muito ruim*	*Ruim*	*Regular*	*Boa*	*Muito boa*	*Excelente*
	**0**	**1**	**2**	**3**	**4**	**5**

36. Liste os medicamentos tomados por seu filho(a) nas últimas 2 semanas:

0. Nenhum medicamento

1. ....

2. ....

3. ....

4. ....

5. ....

6. ....

7. ....

8. ....

9. ....

SEÇÃO 6: QUALIDADE DE VIDA GERAL DE SEU FILHO(A)						
Nas <u>duas últimas semanas</u>	*Muito ruim*	*Ruim*	*Regular*	*Boa*	*Muito boa*	*Excelente*
**37. Como você classificaria a qualidade de vida geral de seu filho(a)?**	**0**	**1**	**2**	**3**	**4**	**5**

SEÇÃO 7: IMPORTÂNCIA DOS ITENS PARA A QUALIDADE DE VIDA DE SEU FILHO(A)

No atual estado de seu filho(a), qual é a importância desses itens na qualidade de vida dele(a)?	*O menos importante*	*Não muito importante*	*Um pouco importante*	*Mais ou menos importante*	*Importante*	*O mais importante*
**1. Comer ou beber ou ser alimentado**	**0**	**1**	**2**	**3**	**4**	**5**
**2. Manter a higiene bucal**	**0**	**1**	**2**	**3**	**4**	**5**
**3.Tomar banho ou lavar-se**	**0**	**1**	**2**	**3**	**4**	**5**
**4. Usar o banheiro ou fazer a higiene**	**0**	**1**	**2**	**3**	**4**	**5**
**5. Trocar fraldas ou roupa de baixo**	**0**	**1**	**2**	**3**	**4**	**5**
**6. Colocar ou tirar roupas da parte de cima do corpo**	**0**	**1**	**2**	**3**	**4**	**5**
**7. Colocar ou tirar roupas da parte de baixo do corpo**	**0**	**1**	**2**	**3**	**4**	**5**
**8. Colocar ou usar calçados**	**0**	**1**	**2**	**3**	**4**	**5**
**9. Cuidar dos cabelos ou se arrumar**	**0**	**1**	**2**	**3**	**4**	**5**
**10. Deitar-se e levantar-se da cama**	**0**	**1**	**2**	**3**	**4**	**5**
**11. Transferir-se para a cadeira de rodas ou cadeira ou sair dela**	**0**	**1**	**2**	**3**	**4**	**5**
**12. Permanecer sentado(a) na cadeira de rodas ou cadeira**	**0**	**1**	**2**	**3**	**4**	**5**
**13. Ficar em pé para se exercitar ou mudar de lugar**	**0**	**1**	**2**	**3**	**4**	**5**
**14. Locomover-se em ambientes fechados**	**0**	**1**	**2**	**3**	**4**	**5**
**15. Locomover-se em ambientes externos**	**0**	**1**	**2**	**3**	**4**	**5**

16. Entrar e sair de veículos	0	1	2	3	4	5
17. Visitar lugares públicos	0	1	2	3	4	5
18. Conforto durante a alimentação	0	1	2	3	4	5
19. Conforto ao usar o banheiro	0	1	2	3	4	5
20. Conforto ao colocar ou tirar roupas	0	1	2	3	4	5
21. Conforto ao se transferir de um lugar para outro ou mudar de posição	0	1	2	3	4	5
22. Conforto enquanto está sentado(a)	0	1	2	3	4	5
23. Conforto enquanto está deitado(a)	0	1	2	3	4	5
24. Conforto enquanto dorme	0	1	2	3	4	5
25. Estado emocional ou comportamento	0	1	2	3	4	5
26. Felicidade	0	1	2	3	4	5
27. Capacidade de compreender você	0	1	2	3	4	5
28. Capacidade de ser compreendido(a) por você	0	1	2	3	4	5
29. Capacidade de comunicar-se com outras pessoas	0	1	2	3	4	5
30. Capacidade de brincar sozinho(a)	0	1	2	3	4	5
31. Capacidade de brincar com outras pessoas	0	1	2	3	4	5
32. Capacidade de frequentar a escola, a creche ou a alfabetização	0	1	2	3	4	5
33. Capacidade de participar de atividades recreativas	0	1	2	3	4	5
34. Minimizar idas ao médico e hospitalização	0	1	2	3	4	5
35. Saúde em geral	0	1	2	3	4	5
36. Minimizar o número de medicamentos	0	1	2	3	4	5

SEÇÃO 8: INFORMAÇÕES SOBRE SEU FILHO(A)

1. Sexo da criança: ( ) Masculino ( ) Feminino

2. Qual é a data de nascimento de seu filho(a)?

______ / ______ / ____________
Dia Mês Ano

3. Qual o maior nível escolar completado por seu filho(a)? (marque apenas um)

( ) Pré-escola	( ) 5º Ano	( ) 9º Ano
( ) 1º Ano	( ) 6º Ano	( ) 1ª Série do Ens. Médio
( ) 2º Ano	( ) 7º Ano	( ) 2ª Série do Ens. Médio
( ) 3º Ano	( ) 8º Ano	( ) 3ª Série do Ens. Médio
( ) 4º Ano		

Frequenta(ou) ensino não seriado? ( )Sim ( )Não. Se sim, há(por) quanto tempo? __________

SEÇÃO 9: IFORMAÇÕES SOBRE VOCÊ

1. Sexo: ( ) Masculino ( ) Feminino

2. Qual é a sua data de nascimento? ______ / ______ / ____________
Dia Mês Ano

3. Qual a melhor descrição de sua situação em relação a trabalho? (marque todas as alternativas que se apliquem)

( ) **Não trabalho atualmente devido à condição de saúde de meu filho(a).**

( ) **Não estou trabalhando por outros motivos.**

( ) **Estou procurando trabalho fora de casa.**

( ) **Trabalho em tempo integral ou meio período, em casa ou fora**

( ) **Sou dona-de-casa em período integral**

4. Qual a melhor descrição de sua relação com a criança?

( ) Pai/mãe biológico (a)	( ) Padrasto/madrasta	( ) Pai/mãe de criação
( ) Pai/mãe adotivo (a)	( ) Tutor	( ) Cuidador Profissional

( ) **Outro (Por favor explique):** ....

**5. Em média, quantos dias por semana você é responsável por cuidar de sua criança?**	**_______ dias por semana**

6. Qual é o seu grau de escolaridade?

**( ) Pré-escola**	**( ) 6º Ano**	**( ) 3ª Série do Ens. Médio**
**( ) 1º Ano**	**( ) 7º Ano**	**( ) Ensino Técnico**
**( ) 2º Ano**	**( ) 8º Ano**	**( ) Ensino Superior**
**( ) 3º Ano**	**( ) 9º Ano**	**( ) Pós-graduação *Lato Sensu***
**( ) 4º Ano**	**( ) 1ª Série do Ens. Médio**	**( ) Pós-graduação *Stricto Sensu***
**( ) 5º Ano**	**( ) 2ª Série do Ens. Médio**	

Frequenta(ou) ensino não seriado? ( )Sim ( )Não. Se sim, há(por) quanto tempo? _______

Quanto tempo você levou para responder <u>somente a este questionário</u> (em minutos):___________________.

OBRIGADO POR SUA PARTICIPAÇÃO!
